# Physiology and methane productivity *of Methanobacterium thermaggregans*

**DOI:** 10.1007/s00253-018-9183-2

**Published:** 2018-06-29

**Authors:** Lisa-Maria Mauerhofer, Barbara Reischl, Tilman Schmider, Benjamin Schupp, Kinga Nagy, Patricia Pappenreiter, Sara Zwirtmayr, Bernhard Schuster, Sébastien Bernacchi, Arne H. Seifert, Christian Paulik, Simon K.-M. R. Rittmann

**Affiliations:** 1 Archaea Physiology & Biotechnology Group, Archaea Biology and Ecogenomics Division, Department of Ecogenomics and Systems Biology, Universität Wien, Althanstraße 14, 1090 Wien, Austria; 2 Krajete GmbH, Linz, Austria; 3 Department of Nanobiotechnology, University of Natural Resources and Life Sciences, Institute of Synthetic Bioarchitectures, Wien, Austria; 4 Johannes Kepler Universität Linz, Institute for Chemical Technology of Organic Materials, Linz, Austria

**Keywords:** Archaea, Methanogen, CH_4_, Biorefinery, Biofuel, Bioprocess, Fed-batch

## Abstract

Accumulation of carbon dioxide (CO_2_), associated with global temperature rise, and drastically decreasing fossil fuels necessitate the development of improved renewable and sustainable energy production processes. A possible route for CO_2_ recycling is to employ autotrophic and hydrogenotrophic methanogens for CO_2_-based biological methane (CH_4_) production (CO_2_-BMP). In this study, the physiology and productivity of *Methanobacterium thermaggregans* was investigated in fed-batch cultivation mode. It is shown that *M. thermaggregans* can be reproducibly adapted to high agitation speeds for an improved CH_4_ productivity. Moreover, inoculum size, sulfide feeding, pH, and temperature were optimized. Optimization of growth and CH_4_ productivity revealed that *M. thermaggregans* is a slightly alkaliphilic and thermophilic methanogen. Hitherto, it was only possible to grow seven autotrophic, hydrogenotrophic methanogenic strains in fed-batch cultivation mode. Here, we show that after a series of optimization and growth improvement attempts another methanogen, *M. thermaggregas* could be adapted to be grown in fed-batch cultivation mode to cell densities of up to 1.56 g L^−1^. Moreover, the CH_4_ evolution rate (MER) of *M. thermaggregans* was compared to *Methanothermobacter marburgensis*, the CO_2_-BMP model organism. Under optimized cultivation conditions, a maximum MER of 96.1 ± 10.9 mmol L^−1^ h^−1^ was obtained with *M. thermaggregans*—97% of the maximum MER that was obtained utilizing *M. marburgensis* in a reference experiment. Therefore, *M. thermaggregans* can be regarded as a CH_4_ cell factory highly suited to be applicable for CO_2_-BMP.

**Electronic supplementary material:**

The online version of this article (10.1007/s00253-018-9183-2) contains supplementary material, which is available to authorized users.

## Introduction

Fossil hydrocarbon utilization has positively promoted our economy and energy infrastructure in the past (Rondinelli and Berry 2000). However, combustion of fossil hydrocarbons is known to adversely affect our health and the environment, and consequently, it contributes to global warming (Hansen et al. 2000). Environmental awareness and decreasing fossil energy sources have driven interests in renewable energy and biofuel production. Biofuels are energy carriers that can be produced from biological resources. They are considered to be eco-friendly. The utilization of biofuels reduces greenhouse gas emissions by recycling waste and carbon dioxide (CO_2_). Biofuel production from agricultural resources, particularly in relation to biological waste, could provide independency from the natural gas exploitation business, both to energy suppliers and to energy end consumers. Moreover, a biofuel-based industry could be integrated into various biorefinery concepts (Martínez-Porqueras et al. 2012). Such an integration could promote the development and application of a circular economy concept by increasing demand and prices for agricultural by-products (Demirbas 2009). As a viable alternative to fossil hydrocarbons, a biofuel should have the following: superior environmental benefits, be sustainably produced, produce a net energy gain over the fossil fuel it is supposed to displace, be available in sufficient quantities (Hill et al. 2006), and be capable of being integrated into the economy of the common goods. Competition between a biofuel source and food production could also be considered, but it is irrelevant if a non-industrialized food production scenario would be globally considered (Muller et al. 2017).

The diversity of currently utilized biofuels belongs to the 1st, 2nd, and 3rd generations (Martínez-Porqueras et al. 2012). However, biofuels from the 4th and 5th generation are under development and only ready at a (pre-) demonstration plant scale. Pure plant oil, hydrotreated vegetable oil (HVO), bioethanol, biomethanol, biodiesel (fatty acid methyl ester (FAME)), biodimethylether, ethyl tert-butyl ether, methyl tert-butyl ether, superethanol E 85, synthetic biofuels, biologically produced molecular hydrogen (H_2_), and biologically produced methane (CH_4_) are either currently in use as biofuels or are under development. The manufacturing of gaseous biofuels may be accomplished through a variety of upcycling processes. These upcycling processes can utilize organic waste from biogas plants (Sasse 1988), gasification of biomass (Benedikt et al. 2017; Mauerhofer et al. 2018), dark fermentation of organic biomass from agricultural residues, agro-industrial and organic municipal wastes (Ghimire et al. 2015), or the recycling of CO_2_. In the context of upcycling processes (biological), H_2_ production is also of ecological and biotechnological interest.

H_2_ has a number of advantages as an energy carrier from a gaseous biofuel utilization perspective. H_2_ can be produced from a variety of resources, e.g., olive husk, municipal solid waste, crop grain residue, plastic waste, pulp and paper waste, and manure slurry. H_2_ also has the advantage of clean combustion; the only combustion products are water vapor and tiny amounts of nitrogen oxides (NO_x_) (Ma et al. 2003). High heating and caloric values can be achieved when using H_2_ as a biofuel and starting an H_2_ engine is easy at low temperatures due to the property of H_2_ remaining in the gaseous state until − 253.15 °C (Table 1) (Ma et al. 2003). Hence, the main drawback of H_2_ is that it comprises a low energy density. To circumvent the storage of this low density gas, H_2_ could be directly converted to CH_4_, which is a much better energy carrier. The already existing natural gas pipeline infrastructure in many parts of the world (Shahidehpour et al. 2005; Carvalho et al. 2009) could feasibly integrate biological CH_4_ and renders it a promising energy carrier for long-term energy storage. CH_4_ can be used as a biofuel, a heating, and cooking fuel, or be reconverted into electricity by burning. The higher heating value of CH_4_ compared to gasoline and diesel oil encourages the usage of this biofuel as an important energy vector (Table 1).

**Table 1:** Physicochemical parameters of common fuels

Fuel	Phase	Heating value	Caloric value	Density	Combustion (raw emissions)	Reference
		kWh/kg	kWh/kg	kg/Nm^3^		
H_2_	Gas 0 °C, 1.013 bar	33.3 (~3 kWh/m^3^_n_)	39.4	0.0899	H_2_O	(Linde Gas GmbH 2007; Paschotta 2017)
	Liquid			0.0708		(Linde Gas GmbH 2007)
CH_4_	Gas 0 °C, 1.013 bar	13.9 (~10 kWh/m^3^_n_)	15.4	0.7175	H_2_O, CO_2_	(Paschotta 2018a)
LNG	Liquid			450		(Dinçer and Zamfirescu 2016)
Gasoline	Liquid	11.4	11.9	720–780	CO_2_, H_2_O, CO, NO_x_, HC	(Paschotta 2018b; Hilgers 2016)
Diesel oil	Liquid	11.9	12.6	820–845	H_2_O, CO_2_, CO, NO_x_, HC, particle (solid components, sulphates), aldehydes	(Hoinkis 2015; Hahne 2011; Hilgers 2016)

The biological conversion of H_2_ involves the reduction of CO_2_ to CH_4_ (Balch et al. 1979; Thauer 1990). This reaction can be performed biologically by using autotrophic and hydrogenotrophic methanogens in pure culture (Bernacchi et al. 2014; Rittmann et al. 2015; Schönheit et al. 1980; Seifert et al. 2014) or with enrichment cultures containing methanogens (Burkhardt and Busch 2013; Savvas et al. 2017; Strübing et al. 2017; Rachbauer et al. 2016, 2017; Rittmann 2015). Methanogenic archaea play a crucial role in the global carbon cycle, as they perform the final step in the mineralization of organic matter under anaerobic conditions if no other H_2_ acceptors (nitrate or sulfate) are present (Jabłoński et al. 2015). Besides the fact that methanogenic archaea possess a significant importance for efficient degradation of organic matter in nature, their metabolic capability for CH_4_ production could be an essential milestone in renewable energy production and storage.

The biological conversion of H_2_ and CO_2_ to CH_4_ is referred to as CO_2_-based biological CH_4_ production (CO_2_-BMP) (Abdel Azim et al. 2017; Bernacchi et al. 2014; Rittmann et al. 2015, 2018). A very high CH_4_ evolution rate (MER) of 945 mmol L^−1^ h^−1^ has been previously obtained in a lab-scale continuous culture CO_2_-BMP system (Seifert et al. 2014). Higher MERs could only be obtained by improving the bioprocessing conditions (Nishimura et al. 1992; Rittmann et al. 2018). A high CH_4_ off-gas concentration exceeding 95 Vol.-% was recently achieved in pure culture (Bernacchi et al. 2016). With respect to the specific CH_4_ production rate (qCH_4_) of a methanogen, it is important if gas-limited or liquid-limited conditions prevail (Bernacchi and Herwig 2016; Rittmann et al. 2018). Gas-limited conditions occur, e.g., if the organisms face H_2_ and/or CO_2_ limitation. Liquid-limited conditions are encountered by an organism if, e.g., trace elements are limiting the growth and/or gas production. However, before a methanogen is investigated in continuous culture mode, it is highly beneficial to utilize closed batch or fed-batch CO_2_-BMP systems for the initial examination of the physiological and biotechnological characteristics of the organism (Abdel Azim et al. 2017; Taubner and Rittmann 2016).

Hitherto, 155 methanogenic strains have been characterized in pure culture (Holmes and Smith 2016). Methanogens possess different substrate preferences. 74.5% of methanogens utilize H_2_/CO_2_, 33% utilize methylated compounds, and 8.5% utilize acetate. The conversion of methylated compounds is rarely accompanied with the ability to utilize H_2_/CO_2_. Unfortunately, the substrate preference of characterized methanogens is still incomplete (Jabłoński et al. 2015). Until now only seven methanogens in fed-batch cultivation mode with constant H_2_/CO_2_ supply have been cultivated. There is a need to understand how methanogens can be grown in fed-batch cultivation mode, to extend the portfolio of methanogens that may be utilized for CO_2_-BMP and for physiological, biochemical, biotechnological, and environmental studies.

In this study, the physiology and CH_4_ productivity of *Methanobacterium thermaggregans* (Blotevogel and Fischer 1985) was investigated using fed-batch cultivation mode. First, CO_2_-BMP of *M. thermaggregans* was examined with respect to inoculation, agitation speed, and sulfur feeding rate. By using this strategy, *M. thermaggregans* could be adapted to grow at high agitation speed. Second, optimization of growth and CH_4_ productivity was performed by using a multivariate statistical optimization procedure. Third, the optimized CH_4_ productivity of *M. thermaggregans* was compared to the CH_4_ productivity of *Methanothermobacter* *marburgensis* in a reference experiment with the most well-characterized CO_2_-BMP microorganism. The aim of this study was to investigate the physiological and biotechnological characteristics of *M. thermaggregans* as well as to assess its application potential in further CO_2_-BMP scale-up endeavors.

## Materials and methods

### Strains

All experiments were performed with the type strain *Methanobacterium thermaggregans* DSM 3266 (Blotevogel and Fischer 1985) and with *Methanothermobacter marburgensis* DSM 2133 (Schönheit et al. 1980). Both strains were obtained from the Deutsche Stammsammlung für Mikroorganismen und Zellkulturen GmbH (DSMZ, Braunschweig, Germany).

### Chemicals

CO_2_ (99.995 Vol.-%), H_2_ (99.999 Vol.-%), and H_2_/CO_2_ (80 Vol.-% H_2_ in CO_2_) were obtained from Air Liquide (Air Liquide GmbH, Schwechat, Austria). All other chemicals were of highest available grade.

### Culture maintenance

Pre-cultures of *M. thermaggregans* were prepared and maintained by using the previously described closed batch cultivation technique (Taubner and Rittmann 2016). The inoculum for all fed-batch cultivations of *M. thermaggregans* and of *M. marburgensis* was obtained from fed-batch cultivations. Harvesting of high cell density biomass was done by using methods previously described (Abdel Azim et al. 2017). All fed-batch cultivations of *M. thermaggregans* and of *M. marburgensis* were performed using *M. marburgensis* medium (MM) (Rittmann et al. 2012).

### Fed-batch cultivations

Fed-batch cultivations of *M. thermaggregans* were performed in parallel with DASGIP® 2.2 L glass bioreactor system (SR1500ODLS, Eppendorf AG, Hamburg Germany). *M. thermaggregans* was cultivated in a volume of 1.5 L MM medium. Individual CO_2_ and H_2_ supply was controlled using separate mass flow controllers. CO_2_ mass flow was controlled via the MX4/4 unit (Eppendorf AG, Hamburg, Germany). H_2_ gas flow was controlled via the C100L unit (Sierra Instruments, Monterey, USA). Before inoculation, and while continuously gassing the bioreactor with H_2_/CO_2_, 3 mL of anaerobically prepared 0.5 mol L^−1^ Na_2_S·9H_2_O were anaerobically added to the bioreactor.

### Initial examination of inoculation volume, agitation and sulfide feed

To investigate growth and CH_4_ productivity of *M. thermaggregans*, bioprocess input variables such as inoculation volume, agitation speed, and Na_2_S·9H_2_O feeding rate (DS) were examined (defined as initial experiments). Three different inoculation volumes of 10, 30, and 50 mL were individually investigated. The inoculation volume was always applied to 1.5 L of pre-heated medium having the required pH and oxidation reduction potential. Initial attempts to cultivate *M. thermaggregans* at agitation speeds of 1000, 1200, or 1600 revolutions per minute (rpm) failed. Therefore, different agitation profiles were tested: 600 rpm throughout the whole cultivation, 4-h rpm ramp from 200 to 1600 rpm, and a 6-h rpm ramp from 200 to 1600 rpm. The intention of the agitation speed ramp was to let *M. thermaggregans* slowly adapt to higher rpm values. DS of 0.2 and 0.6 mL h^−1^, and DS ramps from 0.2–0.6 to 0.6–0.9 mL h^−1^ were tested. The culture was continuously gassed with 0.5 vvm H_2_/CO_2_ during the whole fed-batch cultivation.

### Fed-batch DoE experiments

After performing the initial experiments and to determine optimal inoculation volume, agitation speed, and DS in the fed-batch cultivation mode, a design of experiment (DoE) approach was used to investigate the optimal temperature and pH for growth and CH_4_ productivity of *M. thermaggregans*. A DoE allows the investigation of main factors, e.g., temperature and pH, and their interaction towards the parameter of interest, e.g., growth and CH_4_ production. Furthermore, not only the effects caused by main factors can be quantified, but also their interaction can be analyzed. The data obtained from randomized individual runs are then statistically analyzed. This statistical analysis can describe functional interaction between input factors and the results (Anderson and Whitcomb 2010). The chosen DoE setting compromised 22 randomized runs within a temperature range from 50 to 70 °C and a pH range from 6.2 to 7.8. The pH was controlled by titration using 1 mol L^−1^ HCl or 1 mol L^−1^ NaOH. For every run, 30 mL of inoculum with an optical density (OD) at a wavelength of 578 nm of 5.1 was used. The initial gas flow rate was 0.3 vvm, and consisted of individually controlled 5 L_n_ h^−1^ CO_2_ and 20 L_n_ h^−1^ H_2_. Just before inoculation, 3 mL of 0.5 mol L^−1^ Na_2_S·9H_2_O was anaerobically added to the bioreactor. In addition, 0.5 mol L^−1^ Na_2_S·9H_2_O was continuously added with a DS of 0.3 mL h^−1^. This setting was maintained for 10 h at an agitation speed of 200 rpm, followed by a 4-h ramp from 200 to 1600 rpm. Fifteen hours after inoculation, the gas flow rate was increased to 1 vvm (20 L_n_ h^−1^ CO_2_ and 80 L_n_ h^−1^ H_2_). Henceforth, OD was measured periodically and off-gas samples (Reischl et al. 2018) were taken in 2-h intervals. The experiment lasted for 10 h, after raising the gas flow rate to 1 vvm. If growth stagnation was observed, DS was increased to 0.6 mL h^−1^.

### Exponential fed-batch for comparing the performance of *M. thermaggregans* to *M. marburgensis*

Exponential fed-batch experiments were performed for comparing growth and CH_4_ productivity of *M. thermaggregans* and *M. marburgensis*. Both organisms were grown at their optimal or optimized growth conditions, which were as follows: *M. marburgensis* at 65 °C, pH = 7.0 (Abdel Azim et al. 2017; Bernacchi et al. 2014; Schönheit et al. 1980) and *M. thermaggregans* 60 °C, pH = 7.0 (Blotevogel and Fischer 1985). Thirty milliliters of inoculum with an OD_578nm_ of 5.1 was used for inoculation. Shortly before inoculation, 3 mL of 0.5 mol L^−1^ Na_2_S·9H_2_O was anaerobically added and thereafter a constant DS of 0.1 mL h^−1^ was applied. A H_2_/CO_2_ flow rate of 0.3 vvm was applied for 10 h, followed by a 4-h agitation ramp from 200 to 1600 rpm. Fifteen hours after inoculation, the H_2_/CO_2_ flow rate and the DS were exponentially increased to 1.5 vvm and 0.3 mL h^−1^ within 10 h. OD_578nm_ was spectrophotometrically measured (Beckmann Coulter DU 800 spectrophotometer) and off-gas samples (Reischl et al. 2018) were taken every 2 h.

### Analysis of growth, off-gas composition, and productivity

During all initial experiments, DoE experiments, and exponential fed-batch experiments, growth was quantified by measuring OD_578nm_. Before every OD_578nm_ measurement, the sample was vortexed (Vortex Mixer MX-S, Biologix Group Limited, China). H_2_ and CO_2_ uptake rates and MER were calculated by determining the off-gas composition and calculating or measuring the off-gas volumetric flow rate. The off-gas composition (H_2_, CO_2_, and CH_4_) during cultivation was analyzed via gas chromatography (7890A GC System, Aligent Technologies, Santa Clara, USA) by using a TCD detector and a 19808 Shin Carbon ST Micropacked Column (Restek GmbH, Bad Homburg, Germany) as described before (Taubner and Rittmann 2016; Abdel Azim et al. 2017). Automated sampling of serum bottle headspace and subsequent gas injection into the gas chromatograph for off-gas analysis was accomplished by using a gas injection and control unit (Joint Analytical Systems GmbH, Moers, Germany). In the case of the DoE experiments and additional runs, the off-gas flow rate was measured with a TG3 plastic drum-type gas meter (Ritter GmbH, Bochum, Germany). Therefore, it was necessary to measure the pressure inside the bioreactor by using a digital manometer (LEO1, Keller GmbH, Winterthur, Switzerland). The off-gas temperature was monitored and controlled through the process and information management system.

### Elementary analysis of *M. thermaggregans* biomass

The elementary composition of two separately performed *M. thermaggregans* fed-batch runs was determined. The biomass was pelleted and washed twice with ddH_2_O via centrifugation (3 × 30 min., 24,000*g*, 4 °C, superspeed centrifuge, Sorvall LYNX 4000, Thermo Fisher Scientific, Austria). The pellets were then lyophilized for 24 h (freeze-dryer, Alpha 1-4 LSC, Martin Christ Gefriertrocknungsanlagen GmbH, Germany). The lyophilized sample was homogenized by using a mortar and pestle. 4.7–4.9 mg of the homogenized sample were used to perform an elementary analysis using a Thermo Flash EA 1112 series CHNS Analyzer (Thermo Fisher Scientific, Vienna, Austria). The device was calibrated with BBOT standard (2, 5-Bis (5-ter-butyl-benzoxazol-2-yl) thiophene). The elementary composition of *M. thermaggregans* biomass was CH_1.8698_N_0.2184_O_0.4529_S_0.0002_. The molar weight of biomass of *M. thermaggregans* was calculated as 29.10 g C-mol^−1^ (normalized to 1 mol of carbon). The ash content was 16.83% ± 0.68. Therefore, the degree of reduction (DoR) was 4.31 e^−^ C-mol^−1^. The C-molar weight of the biomass and the DoR were used to calculate carbon- and DoR-balances.

### Data analysis

To analyze quantitative data from *M. thermaggregans* or *M. marbur*gensis fed-batch cultivations, the following variables were determined or calculated: biomass (*X* [g]), biomass concentration (*x* [g L^−1^]), biomass production rate (*r*_(x)_ C-mmol g^−1^ h^−1^]). *X* and *x* were ascertained via multiplication of OD_578nm_ values and an experimentally determined correlation factor (0.31), which is used to correlate OD_578nm_ measurements and cell dry weight in a linear range (Taubner and Rittmann 2016). To investigate CH_4_ production kinetics, volumetric CH_4_ evolution rate (MER mmol L^−1^ h^−1^]) (volumetric CH_4_ productivity), cumulative (cum.) CH_4_ production [mmol], cum. CH_4_ productivity_max_ [mmol h^−1^], and maximum specific CH_4_ production rate (qCH_4,max_ [mmol g^−1^ h^−1^], qCH_4_ = MER/*x*) were calculated. MER was calculated either by using the *r*_inert_ correction factor (Rittmann et al. 2012; Seifert et al. 2014) and CH_4_ off-gas concentration, or directly from measuring the off-gas flow rate with a drum-type gas meter and CH_4_ off-gas concentration.

### Data analysis of fed-batch initial experiments

The MER_average_ was calculated from all runs performed at the same conditions. For the calculation of MER_max_, cum. CH_4_ productivity_max_ and qCH_4,max_, the max. values of designated runs (equal process parameters), were averaged. The cum. CH_4_ productivity illustrates the cum. CH_4_ production divided by cultivation time. The observation numbers of experiments are shown in Table 2.

**Table 2:** Observation number (*n*) of initial set-up experiments in fed-batch cultivation mode

Fed-batch pre-experiments	MER_average_	MER_max_	cum. CH_4_ productivity_max_	qCH_4,max_
Inoculation volume				
10 mL	6	2	2	2
30 mL	4	2	2	2
50 mL	9	3	3	3
Agitation				
600 rpm	6	2	2	2
4-h ramp (200–1600 rpm)	10	2	2	2
6-h ramp (200–1600 rpm)	7	2	2	2
DS				
0.2 mL h^−1^	3	1	1	1
0.6 mL h^−1^	30	7	7	7
0.2–0.6 mL h^−1^	19	6	6	6
0.6–0.9 mL h^−1^	17	5	5	5

### Data analysis of fed-batch DoE experiments

Statistical analyses of the DoE experiments were performed using analysis of variance (ANOVA) to identify correlations between different process input and output variables using Design Experts® Version 11 (State-Ease Inc., Minneapolis, USA). The models for the prediction of optimal cultivation temperature and pH of *M. thermaggregans* from fed-batch cultivation mode were obtained by fitting input and output variables. Visualization of variable fitting was obtained by using response surface plots. The model significance was estimated from the *p* value and the lack of fit. Model validity was assessed by using *R*^2^. Adjusted *R*^2^ and predicted *R*^2^ have to be similar. The adequate precision (signal to noise ratio) should be above 4. If the evaluation of all those parameters was found to be acceptable, the significance of the prediction model was validated. Twenty-one fed-batch experimental runs out of the 22 performed runs were used for data analysis and model generation. The chosen DoE setting compromised 18 runs and 4 additional runs (K, L, P, and T) with a temperature range from 50 to 70 °C and a pH range from 6.2 to 7.8. Both the data from the DoE runs and the additional runs were used for data analysis to strengthen the prediction of the model. Run H was discarded from the data analysis because it was determined to be outside the three-sigma interval. The optimal temperature and pH of *M. thermaggregans* under the given conditions were determined by using the model calculations. A predicted optimum was then calculated. The optimum was calculated by using a temperature range form 50–70 °C in 1 °C steps, and a pH range form 6.2–7.8 in 0.1 increments. Response surface plots of respective models were generated to depict the relationship that exists between the input and output experimental matrices for the selected key process parameters.

### Data analysis of exponential fed-batch for comparing the growth and productivity of *M. thermaggregans* to *M. marburgensis*

The growth and productivity of *M. thermaggregans* without exponential feeding of H_2_/CO_2_ and DS was compared to *M. marburgensis* fed-batch cultivations. The exponential feeding experiments were performed at 65 °C and a pH of 7.0 for *M. marburgensis*, while for *M. thermaggregans*, the temperature and pH were controlled at 60 °C and 7.0 respectively*.* The maximal values for cum. CH_4_ production, MER_max_, *r*_(x),max_, and *x*_max_ of non- and exponential fed-batch runs were averaged. The maximal values for non-exponential runs were obtained from DoE experiments (cum. CH_4_ production: 60 °C and a pH of 7.0, MER_max_: 65 °C and a pH of 7.4, and *r*_(x),max_ and *x*_max_ at 60 °C and a pH of 7.8).

## Results

### Initial examination of inoculation volume, agitation, and sulfide feed

**Fig. 1:**
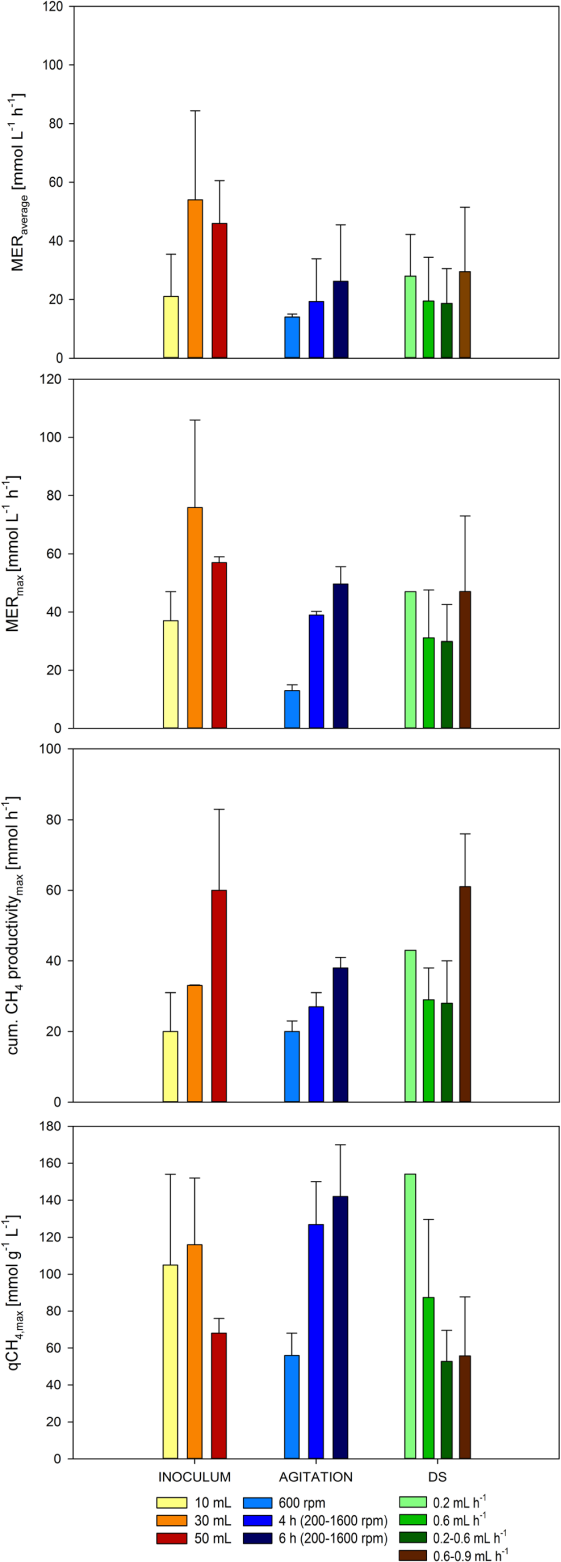
Results of average and max. CH_4_ evolution rate (MER_average_ and MER_max_), max. cumulative CH_4_ production (cum. CH_4_ productivity_max_), and max. specific CH_4_ production rate (qCH_4,max_) for different conditions of inoculation volume, agitation speed, and DS during fed-batch cultivations of *M. thermaggregans* are illustrated. The results of tested inoculation volumes, described as inoculum in the figure, are shown by yellow, orange, and red bars. The tested agitation speed and the two agitation ramps mentioned as agitation in the figure are shown by blue colored bars. Green and brown bars indicate the results of tested DS. All fed-batch cultivation were performed at 65 °C, within 1.5 L of MM medium, and continuously gassed with 0.5 vvm H_2_/CO_2_ (80 Vol.-% H_2_ in CO_2_) at atmospheric pressure. The observation numbers are shown in Table 2

A fed-batch pre-screening with the aim to examine biological CH_4_ production and growth of *M. thermaggregans* with key process parameters such as inoculation volume, agitation speed, and DS was performed. MER_average_, MER_max_, cum. CH_4_ productivity_max_, and qCH_4,max_ (Fig. 1) were examined. First, three different inoculation volumes (Fig. 1: yellow, orange, and red bars) of *M. thermaggregans* suspension were investigated. The highest MER_average_ of 54 ± 30 mmol L^−1^ h^−1^, MER_max_ of 76 ± 30 mmol L^−1^ h^−1^, and qCH_4,max_ of 116 ± 36 mmol g^−1^ h^−1^ were obtained by applying 30 mL of culture (Fig. 1**)**. The highest cum. CH_4_ productivity_max_ of 63 ± 23 mmol h^−1^ was obtained by using 50 mL culture. Based on the results shown in Fig. 1, 30 mL of *M. thermaggregans* cell suspension of an OD_578nm_ = 5.13 contained sufficient biomass to function optimally as a biocatalyst for subsequent fed-batch cultivations.

To examine how a high CH_4_ production with *M. thermaggregans* could be achieved in fed-batch cultivation mode, different rpm settings were tested to understand the tolerance of sheer stress that originated from agitation. The results of the experiments with three different agitation regimes (Fig. 1: blue bars) of 600 rpm over the whole cultivation, beginning with a 4-h ramp from 200 to 1600 rpm or a 6-h ramp from 200 to 1600 rpm, are shown in Fig. 1. The highest MER_average_ of 26 ± 19 mmol L^−1^ h^−1^, MER_max_ of 50 ± 6 mmol L^−1^ h^−1^, cum. CH_4_ productivity_max_ of 38 ± 3 mmol h^−1^, and qCH_4,max_ of 142 ± 28 mmol g^−1^ h^−1^ were obtained by applying a 6-h ramp from 200 to 1600 rpm. However, it was observed that *M. thermaggregans* did not grow when an agitation speed of 1000, 1200, 1400, or 1600 rpm was applied directly from the beginning of the fed-batch cultivations (data not shown). *M. thermaggregans* can be reproducibly adapted to grow at a high agitation speed (1600 rpm). However, this methanogenic archaeon is not able to grow if a high agitation speed is applied directly after inoculation.

In order to elucidate the optimal sulfur feed, DS values of 0.2 and 0.6 mL h^−1^ or DS ramps from 0.2–0.6 or 0.6–0.9 mL h^−1^ were tested. In Fig. 1 (green and brown bars), the results of *M. thermaggregans* growth and CH_4_ productivity during varying DS are shown. Although average and maximum CH_4_ evolution rate (MER_average_ and MER_max_) values at a DS of 0.2 mL h^−1^ were similar to those MER values that were obtained by using a DS ramp from 0.6 to 0.9 mL h^−1^, the highest MER_average_ of 29 ± 22 mmol L^−1^ h^−1^, MER_max_ of 47 ± 26 mmol L^−1^ h^−1^, and cum. CH_4_ productivity_max_ of 61 ± 15 mmol h^−1^ were obtained by applying a DS ramp from 0.6 to 0.9 mL h^−1^. A maximum specific CH_4_ production rate (qCH_4,max_) of 77 mmol g^−1^ h^−1^ was achieved in a single experiment by applying a DS of 0.2 mL h^−1^.

### Fed-batch DoE experiments

**Fig. 2:**
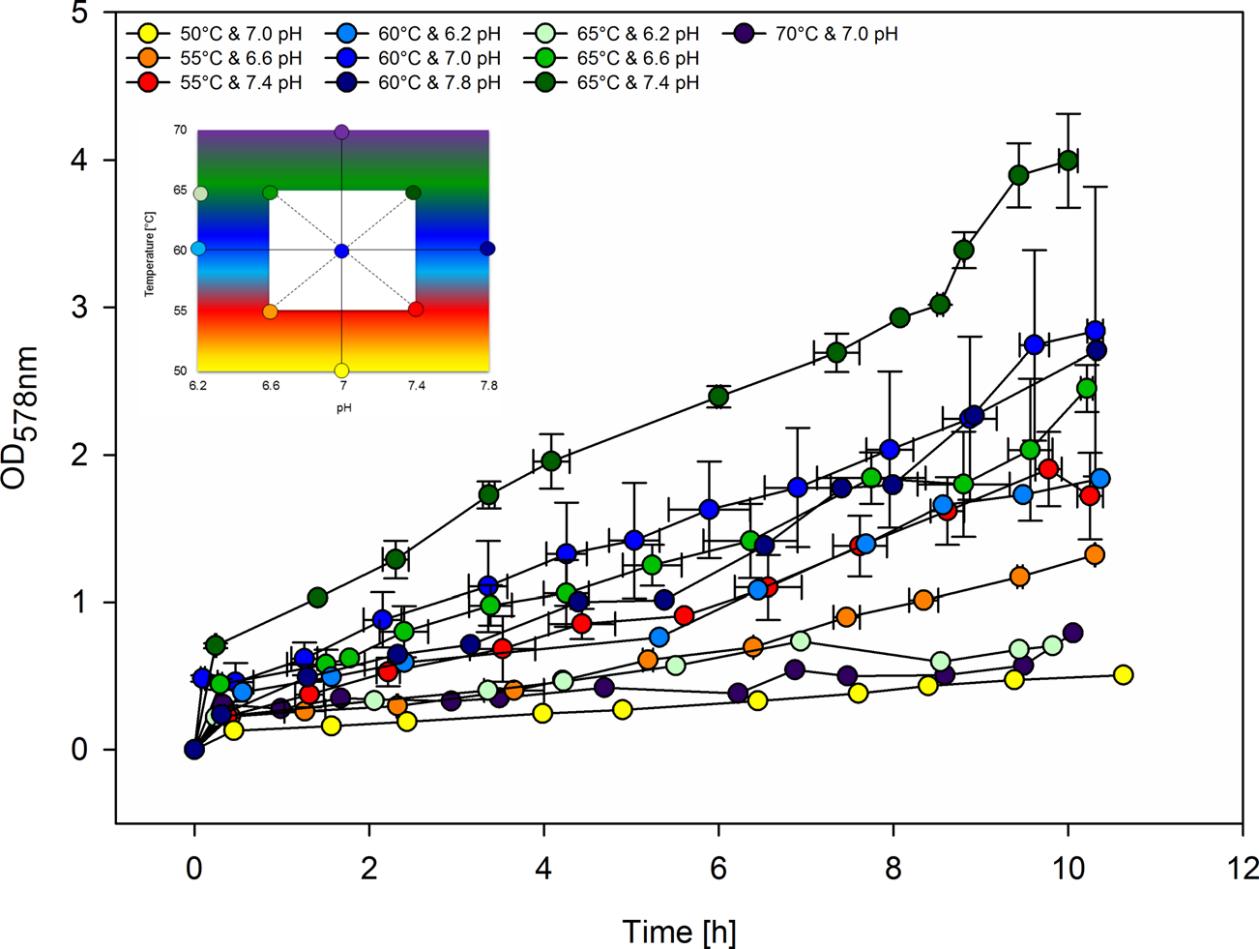
DoE raw data growth curves showing OD_578nm_ values plotted as function of time. The DoE was based on a central composite design (figure in the upper left corner). Temperature and pH were systematically varied in a multivariate design space to determine the optimal cultivation temperature and pH. Experiments indicated with yellow (50 °C and 7.0 pH), light blue (60 °C and 6.2 pH), dark blue (60 °C and 7.8 pH), light green (65 °C and 6.2 pH), and violet (70 °C and 7.0 pH) dots were performed once. Experiments illustrated with orange (55 °C and 6.6 pH), red (55 °C and 7.4 pH), and dark green (65 °C and 7.4 pH) dots were performed twice. Experiments shown with green dots (65 °C and 6.6 pH) were performed in triplicates. The center point (blue dot in the middle of the white box) was examined in octuplicates. The colors of the dots of the figure in the upper left corner correspond to the growth curves. The different colors represent different cultivation conditions

**Fig. 3:**
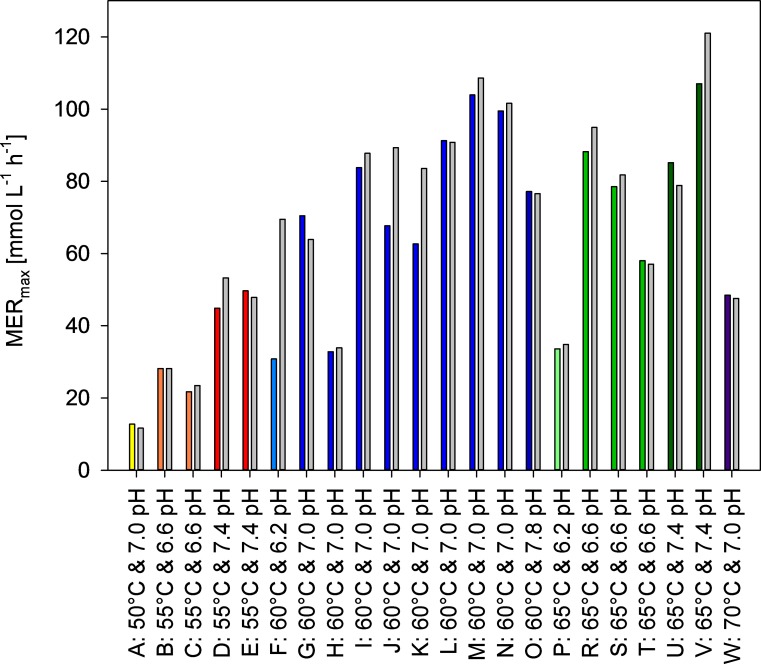
Maximum CH_4_ evolution rate (MER_max_) values from the DoE fed-batch experiment of *M. thermaggregans* shown as a function of temperature and pH. MER_max_ values were calculated via gas outflow correction factor, referred to as *r*_inert_ (gray bars) or through off-gas measurements by using a drum-type gas meter (colored bars). All DoE fed-batch cultivations were performed within a temperature range from 50 to 70 °C and pH range from 6.2 to 7.8. *M. thermaggregans* was cultivated within 1.5 L of MM medium and continuously gassed with 1 vvm H_2_/CO_2_ (80 Vol.-% H_2_ in CO_2_) at atmospheric pressure. In addition, 0.5 mol L^−1^ Na_2_S·9H_2_O was continuously added with a DS of 0.3 mL h^−1^. Experiments indicated with a yellow (A: 50 °C and 7.0 pH), light blue (F: 60 °C and 6.2 pH), dark blue (O: 60 °C and 7.8 pH), light green (P: 65 °C and 6.2 pH), and violet (W: 70 °C and 7.0 pH) bar were performed once. Experiments illustrated with an orange (B, C: 55 °C and 6.6 pH), red (D, F: 55 °C and 7.4 pH), green (R, S: 65 °C and 6.6 pH), and dark green (U, V: 65 °C and 7.4 pH) bars were performed twice. Experimental results shown with green bars (R, S, T: 65 °C and 6.6 pH) were performed in triplicates. The center point indicated with blue bars (60 °C and 7.0 pH) was examined in octuplicates

After defining an appropriate pre-culture volume for inoculation, a procedure to control agitation speed after inoculation, and a suitable rate for DS during fed-batch of *M. thermaggregans*, a multivariate optimization was performed to identify the optimal pH and temperature for growth and CH_4_ production. These two additional key process parameters were investigated in a DoE setting that compromised 18 runs and 4 additional runs (K, L, P, and T). In this DoE, the range for temperature from 50 to 70 °C was selected while the pH ranged from 6.2 to 7.8 (Fig. 2). Both the data from initial DoE screening runs and the additional runs were used for the final model generation. To further substantiate the CH_4_ productivity results, the reproducibility of the total gas outflow rate calculated from the outflow correction factor (*r*_inert_) (Bernacchi et al. 2014; Rittmann et al. 2012; Seifert et al. 2013, 2014) was compared to the experimentally measured off-gas flow rate using a drum-type gas meter. Hence, MER_max_ values were calculated in two ways: from *r*_inert_ and CH_4_ off-gas concentration, or from the total gas outflow and CH_4_ off-gas concentration. Figure 3 illustrates the results of these calculations. Most MER_max_ calculations resulted in similar values with the exceptions being DoE runs D, F, G, J, K, and V which deviated by more than 10%. Results of ANOVA indicated that *r*_inert_ MER_max_ showed a higher *R*^2^ (0.92), adjusted *R*^2^ (0.90), predicted *R*^2^ (0.85), and signal to noise ratio (24.1) when compared to the total gas outflow MER_max_ calculations comprising an *R*^2^ of 0.87, an adjusted *R*^2^ of 0.84, a predicted *R*^2^ of 0.73, and a signal to noise ratio of 19.5, see Tables S3 and S4. Moreover, the model standard deviation was lower (0.0804) from the *r*_inert_ MER_max_ calculations when compared to the model standard deviation (0.1024) from total gas outflow MER_max_ calculations (compare Tables S3 and S4). Therefore, the multivariate statistical analyses were subsequently based on *r*_inert_ MER_max_ calculations.

**Fig. 4:**
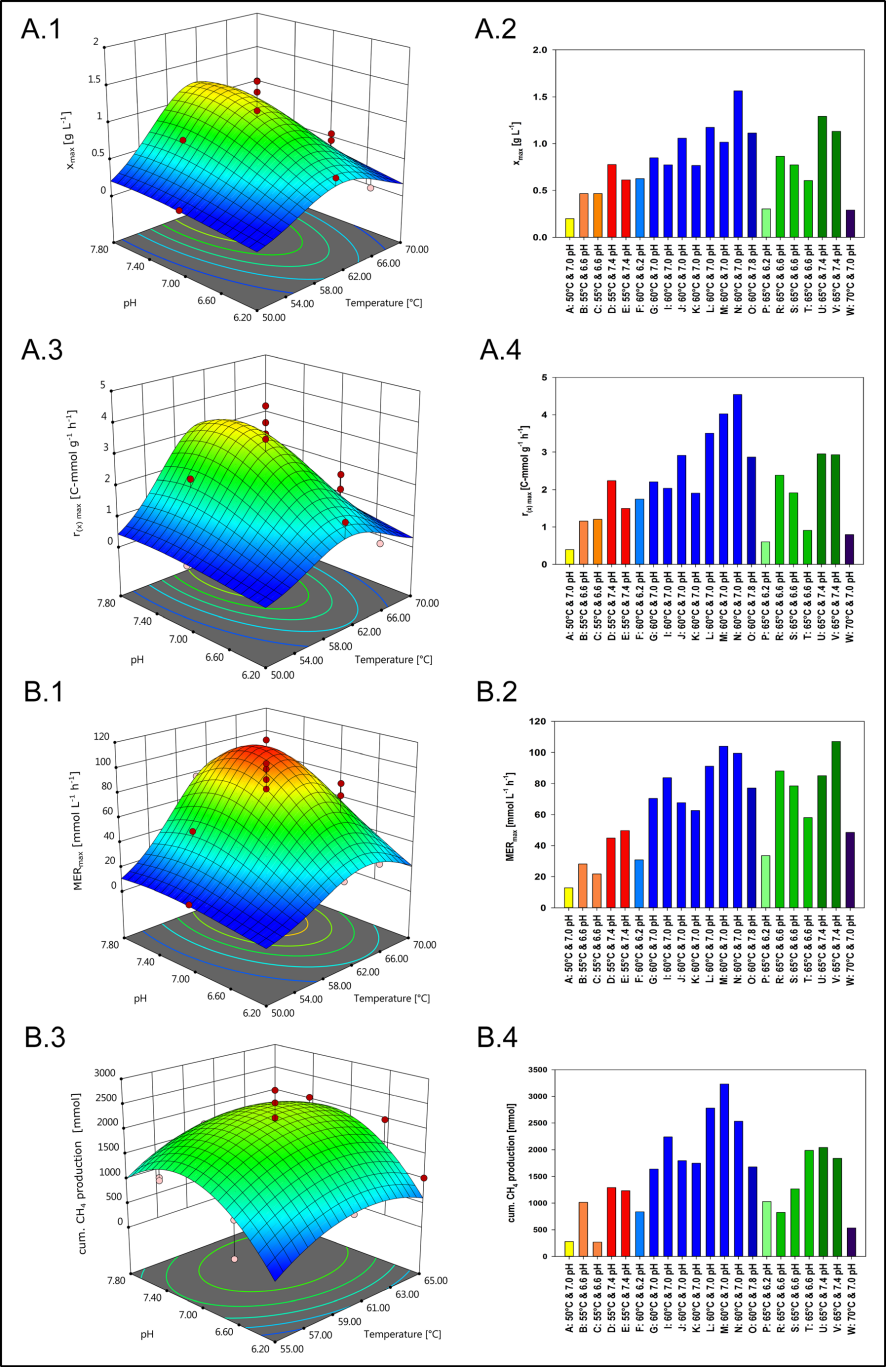
Response surface plots and individual results of growth and CH_4_ productivity of *M. thermaggregans* are shown as functions of temperature (50–70 °C) and pH (6.2–7.8). In **A.1**, **A.3**, **B.1**, and **B.3**, four surface response plots are shown for maximum biomass concentration (*x*_max_), maximum biomass production rate (*r*_(x),max_), maximum CH_4_ evolution rate (MER_max_), and cumulative CH_4_ production (cum. CH_4_ production), respectively. In **A.2**, **A.4**, **B.2**, and **B.4**, the individual results corresponding to the response surface plots for *x*_max_, *r*_(x),max_, MER_max_, and cum. CH_4_ production are illustrated. *M. thermaggregans* was cultivated within 1.5 L of MM medium and continuously gassed with 1 vvm H_2_/CO_2_ (80 Vol.-% H_2_ in CO_2_) at atmospheric pressure. In addition, 0.5 mol L^−^^1^ Na_2_S·9H_2_O was continuously added with a DS of 0.3 mL h^−^^1^. Experiments indicated with yellow (A: 50 °C and 7.0 pH), light blue (F: 60 °C and 6.2 pH), dark blue (O: 60 °C and 7.8 pH), light green (P: 65 °C and 6.2 pH), and violet (W: 70 °C and 7.0 pH) bars were performed once. Experiments illustrated with orange (B, C: 55 °C and 6.6 pH), red (D, F: 55 °C and 7.4 pH), green (R, S: 65 °C and 6.6 pH), and dark green (U, V: 65 °C and 7.4 pH) bars were performed twice. Experimental results shown with green bars (R, S, T: 65 °C and 6.6 pH) were performed in triplicates. The center point indicated with blue bars (G–N: 60 °C and 7.0 pH) was examined in octuplicates

To further perform a physiological comparison to other yet characterized hydrogenotrophic, autotrophic methanogens, growth and CH_4_ production were examined with respect to pH and temperature in fed-batch cultivation mode. The response surface pots visualizing the models are shown in Fig. 4(A.1, A.3, B.1, and B.3). Original results are shown as bar graphs in Fig. 4(A.2, A.4, B.2, and B.4). The ANOVA tables *x*_max_ (Table S1), *r*_(x),max_ (Table S2), MER_max_ (Table S3), and cum. CH_4_ production (Table S5) are shown in the Supplementary material. *x*_max_, *r*_(x),max_, MER_max_, and cum. CH_4_ production are shown as functions of pH and temperature in the response surface models and in the individual results (Fig. 4). The response surface plot of Fig. 4(A.1) suggests an optimal temperature and pH of 61 °C and 7.5 respectively for *x*_max_. In Fig. 4(A.3), an optimal temperature of 61 °C and pH of 7.4 is illustrated for *r*_(x),max_. The response surface plot shown in Fig. 4(B.1) indicates an optimal temperature of 63 °C and a pH of 7.3 for MER_max_. A temperature and pH optimum for cum. CH_4_ production were predicted at 61 °C and 7.2 respectively (Fig. 4(B.3)). When plotting all individual results of cum. CH_4_ production, an optimum temperature of 60 °C and pH of 7.0 are displayed (Fig. 4(B.4)). The pH optimum could be narrowed down to 7.3 to 7.5 when *x*_max_, *r*_(x),max_, and MER_max_ are considered (Fig. 4(A.1, A.3, and B.1)). Moreover, it can be seen that the optimum concerning growth and CH_4_ production (61 °C) (compare Fig. 4(A.1, A.3) to Fig. 4(B.1)) and ANOVA results shown in the supplementary material (Tables S1, S2, S3, and S5) are slightly shifted to a higher temperature (63 °C) for MER_max_ (Fig. 4(B.1)). These results show that *M. thermaggregans* is a slightly alkalophilic, thermophilic methanogen.

### Exponential fed-batch for comparing the performance of *M. thermaggregans* to *M. marburgensis*

**Fig. 5:**
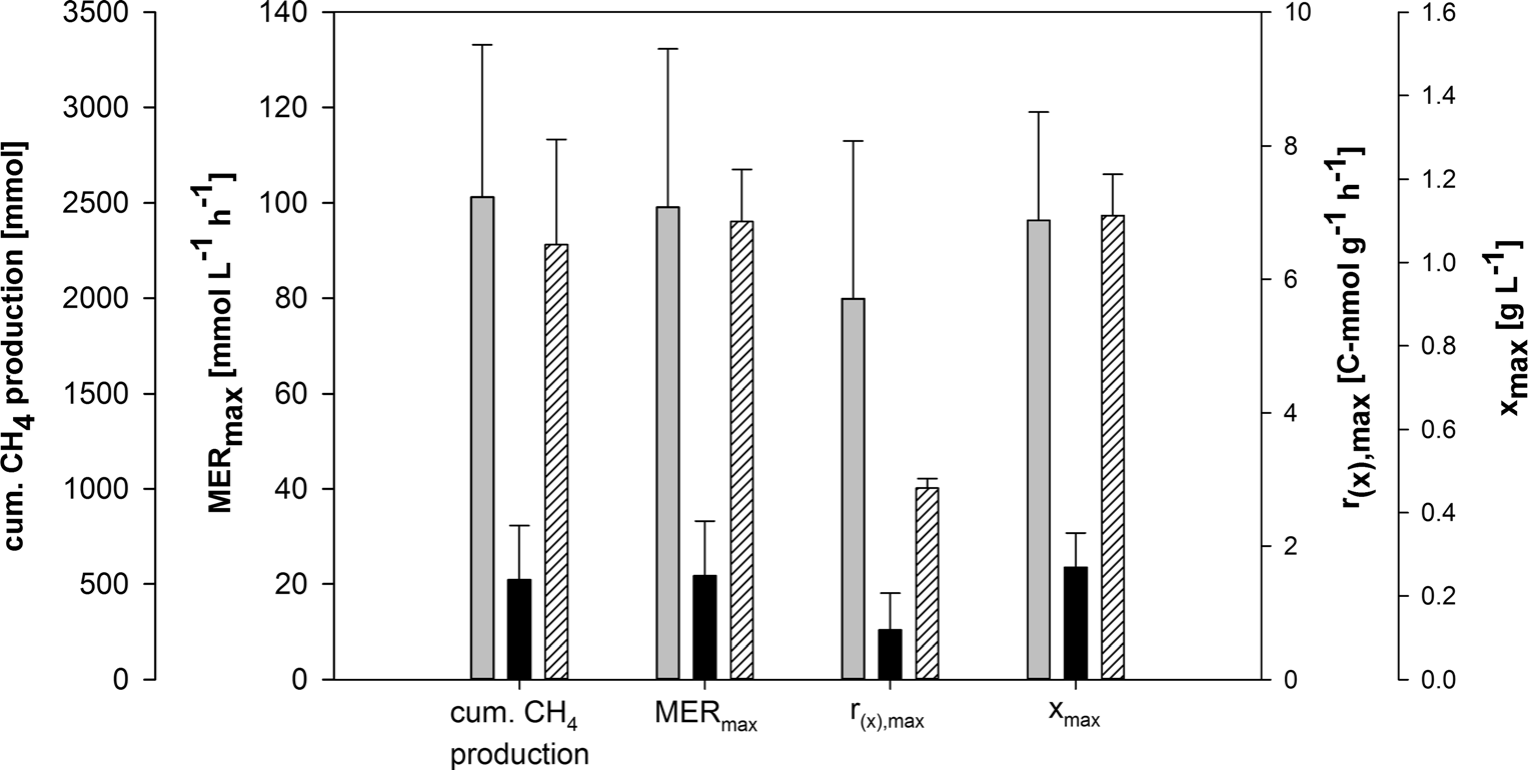
Comparison of cumulative CH_4_ production (cum. CH_4_ production), max. CH_4_ evolution rate (MER_max_), max. biomass production rate (*r*_(x),max_), and maximum biomass concentration (*x*_max_) of *M. thermaggregans* and *M. marburgensis*. The gray bars indicate the performance of *M. marburgensis* at 65 °C, a pH of 7.0, and with exponential feed. The black bars show *M. thermaggregans* cultivated at 60 °C, a pH of 7.0, and with exponential feed. Both strains were cultivated within 1.5 L of MM medium and continuously gassed with H_2_/CO_2_ (80 Vol.-% H_2_ in CO_2_) at atmospheric pressure. H_2_/CO_2_ and DS were exponentially fed to the suspension. The exponential feeding experiments were performed in triplicates. Striped bars show the results from *M. thermaggregans*, observed at the following conditions (optimal DoE runs): cum. CH_4_ production (G–N: 60 °C and 7.0 pH), MER_max_ (U, V: 65 °C and 7.4 pH), and *r*_(x),max_ and *x*_max_ (O: 60 °C and 7.8 pH)

Three different settings of growth and CH_4_ production for either *M. thermaggregans* or *M. marburgensis* were compared. First, *M. marburgensis* was grown at 65 °C and a pH of 7.0 with exponential feeding of gas and sulfur (Abdel Azim et al. 2017). Second, *M. thermaggregans* was grown at 60 °C and a pH of 7.0 with exponential gas and sulfur feeding. Third, the results from the *M. thermaggregans* DoE experiments under the corresponding optimal growth conditions were also considered. The results of cum. CH_4_ production, MER_max_, *r*_(x),max_, and *x*_max_ from the first two experiments and from the DoE results (striped bars) are shown in Fig. 5. Under exponential H_2_/CO_2_ and DS, cum. CH_4_ production, MER_max_, and *x*_max_ values of *M. thermaggregans* depict on average only one fifth of the corresponding values obtained with *M. marburgensis*. *r*_(x),max_ only reached approx. one eighth of the *M. marburgensis* values. Under the respective optimal growth and CH_4_ productivity conditions, these cultures reached a gas-limited state. However, under the optimized growth conditions, *M. thermaggrgans* revealed a MER of 96.1 ± 10.9 mmol L^−1^ h^−1^, which is 97% of the MER that was obtained with *M. marburgensis*. Based on those results, *M. thermaggregans* and *M. marburgensis* are equally suited to be employed as CH_4_ cell factories.

## Discussion

A targeted optimization of biological CH_4_ production from H_2_/CO_2_ was performed utilizing *M. thermaggregans*. For the first time, the physiology and productivity of *M. thermaggregans* was investigated in fed-batch cultivation mode at atmospheric pressure. Considering that up to now it was only possible to cultivate eight autotrophic, hydrogenotrophic methanogenic strains in fed-batch cultivation mode, including *M. thermaggregans*, our analysis is of physiological and of biotechnological relevance. In Table 3, some biotechnologically and physiologically relevant characteristics of methanogens (MER and qCH_4_) that were already examined in fed-batch mode are shown. The highest MER values were achieved during fed-batch cultivations with *M. marburgensis*. The reported MER values of *Methanothermobacter thermautotrophicus* Hveragerdi are similar to those of *M. thermaggregans*. However, the highest qCH_4_ values from fed-batch cultivations are now indicated for *M. thermaggregans*. Considering that growth and CH_4_ production of *M. marburgensis* was optimized for many years, the concise results presented for growth and CH_4_ production of *M. thermaggregans* in this study indicate that this methanogen is a promising CH_4_ cell factory as it already reached 97% of the MER_max_ of *M. marburgensis*.

**Table 3:** Summary of cultivated methanogenic archaea in fed-batch mode under H_2_/CO_2_ feed

Order	Genus	Species	Strain designation	DSM	Temperature [°C]	MER [mmol L^−^1 h^−^1]	qCH_4_ [mmol g^−^1 L^−^1]	Reference
*Methanobacteriales*	*Methanobacterium*	*bryantii*	M.o.H.G.	862	37	–	136*	(Heine-Dobbernack et al. 1988)
*Methanococcales*	*Methanococcus*	*maripaludis*	JJ	2067	37	–	–	(Shieh and Whitman 1988)
*Methanobacteriales*	*Methanothermobacter*	*thermautotrophicus*	Hveragerd	3590	60	114	24	(Gerhard et al. 1993)
*Methanobacteriales*	*Methanothermobacter*	*thermautotrophicus*	Delta H	1053	65	66	120	(de Poorter et al. 2007; Rittmann et al. 2015; Morgan et al. 1997)
*Methanobacteriales*	*Methanothermobacter*	*marburgensis*	Marburg	2133	65	476.5	176	(Abdel Azim et al. 2017)
*Methanococcales*	*Methanothermococcus*	*okinawensis*	IH1	14208	65	24	124	(Abdel Azim et al. 2017)
*Methanococcales*	*Methanocaldococcus*	*jannaschii*	JAL-1	2661	85	–	–	(Mukhopadhyay et al. 1999)
*Methanobacteriales*	*Methanobacterium*	*thermaggregans*		3266	63	107	236	This study

*Protein content was assumed to be 50% of cell dry weight

It can only be speculated as to why many methanogens were not or could not be grown in fed-batch cultivation mode. Maybe there was not the biotechnological perspective to apply methanogens for CO_2_-BMP, or possibly the shear forces in the stirred tank bioreactors inhibited growth of these organisms. Another point concerns the discrepancy when encountering the cultivation of mesophilic and thermophilic methanogens, as until now mainly thermophilic methanogens were cultivated in fed-batch mode (Table 3). Although, the cultivation of mesophilic methanogens would have some advantages, like higher solubility of gasses, it was not yet systematically examined as to why thermophilic methanogens seem to be much easier to be grown in bioreactors. It could be that most mesophilic methanogens live in close association/symbiosis with eukaryotic organisms. This circumstance can impede the cultivation of mesophilic methanogens, if the growing conditions cannot be well mimicked. Compared to mesophilic methanogens, thermophilic methanogens have mostly been isolated from environments where the dependence on a host is not necessary (Bellack et al. 2011; Takai et al. 2002; Ding et al. 2010; Schönheit et al. 1980). In such environments, the range of available nutrients might be different. Therefore, generally speaking, the nutrition requirements of thermophilic methanogens could be reduced or more specific towards particular substrates. Methanogens are mainly cultured to high growth rates for their subsequent biochemical or physiological examination and for the purpose of investigating their biotechnological potential.

In an industrial context, only cost-efficient media are applied to culture methanogens. Methanogens with a broader nutrition requirement shall hence not be considered for optimization if complex or expensive medium compounds are necessary for growth, or if the organism comprises a fastidious growth behavior. Moreover, fed-batch cultivations allow for resolving the need for nutrients in a shorter time. This is why fed-batch cultivations are of interest from a bioprocess development point of view. Then medium optimization studies, or the analysis of liquid limitation and/or uptake of key substrates, can be performed, given that the proper process analytical technology is applied (Rittmann et al. 2018). There is definitely a need to understand how methanogens can be grown in fed-batch cultivation mode in order to extend the portfolio of methanogens that may be utilized for biochemical, molecular biological, and physiological studies, as well as for CO_2_-BMP. Herein, a strategy for adapting *M. thermaggregans* to high agitation speeds is shown, that could possibly be employed to assist in establishing fed-batch cultivations of other methanogens.

For the first time, growth and biological CH_4_ production of *M. thermaggregans* was successfully optimized (inoculation volume, agitation speed, DS) in fed-batch cultivation mode in stirred tank bioreactors. Concurrently, a suitable and reproducible inoculation procedure for further experimental investigation was defined. Based on the results provided in Fig. 1, it is shown that 30 mL of *M. thermaggregans* cell suspension at an OD_578_ = 5.13 was found to contain sufficient biomass that is optimally suited to be used as a biocatalyst for subsequent fed-batch cultivations. However, the highest cum. CH_4_ productivity_max_ was obtained by using 50 mL of culture. This discrepancy could possibly be explained by a very high CH_4_ productivity obtained in a short period of time or by an overall high CH_4_ production over the whole cultivation period. To examine how a high CH_4_ production with *M. thermaggregans* could be achieved in fed-batch cultivation mode, different rpm settings were tested to understand the tolerance of shear stress that originated from the agitation. A higher agitation speed leads to increased mass transfer and therefore correlates with higher gaseous substrate availability in the liquid phase (Rittmann et al. 2015, 2018; Seifert et al. 2014). Hence, *M. thermaggregans* can be reproducibly adapted to grow at a high agitation speed. However, *M. thermaggregans* cannot directly be grown at a high agitation speed. The question remains as to why *M. thermaggregans* reproducibly requires an adaption phase to a high agitation speed? Possibly, the organism needs to modify the lipid composition of its cytoplasmic membrane or to modify other parts of the cell envelope structure to be able to tolerate high shear forces.

As iron-sulfur clusters are abundant in many enzyme complexes in the Wood-Ljungdahl pathway, most methanogens require external sulfur sources for the synthesis of these complexes (Abdel Azim et al. 2017; Thauer 1990; Thauer et al. 2008). As a side effect, DS also leads to the reduction of chemical compounds in the medium. The highest CH_4_ productivity was obtained by applying a DS ramp from 0.6 to 0.9 mL h^−^1 (Fig. 1**)**. MER_average_ and MER_max_ values at a DS of 0.2 mL h^−1^ were similar to those MER values that were obtained by using a DS ramp from 0.6 to 0.9 mL h^−1^. This could be an indication that the activity of the biocatalyst to produce CH_4_ (MER) is not inhibited, but that *x* is affected. Further indication for liquid limitation can be seen in Fig. 2, as only linear growth was observable. The applied DS was possibly too high, which might have resulted in a complexation of trace elements. Moreover, based on a recent finding that even low DS of 0.09 day^−1^ is sufficient to obtain the highest MER values of 949 to 953 mmol L^−1^ h^−1^ (Rittmann et al. 2018), it is possible that even the lowest tested DS were already high enough to achieve a high CH_4_ productivity.

To be able to perform a physiological comparison to other yet characterized hydrogenotrophic, autotrophic methanogens, growth and CH_4_ production were examined with respect to pH and temperature in fed-batch cultivation mode. Optimization of *M. thermaggregans* growth conditions was successfully performed and physiological variables were for the first time comprehensively modeled. Initially, this organism was described to grow optimally at 65 °C and a pH from 7.0 to 7.4 in closed batch cultivation mode (Blotevogel and Fischer 1985). Based on the obtained results, the pH optimum could be narrowed down to 7.3 to 7.5 when *x*_max_, *r*_(x),max_, and MER_max_ are considered. Moreover, it can be seen that the optimum concerning growth and CH_4_ production are slightly shifted to lower temperature (63 °C) for high MER_max_. However, in general, the optimum growth temperature is found to be lower compared to the results that were obtained for closed batch cultivation mode (Blotevogel and Fischer 1985). According to results of this study, *M. thermaggregans* is a slightly alkaliphilic, thermophilic, CH_4_ producing microorganism. Furthermore, the comparison between measured and calculated gas outflow revealed that the *r*_inert_ correction factor is an approximation tool to determine MER from fed-batch cultivations. Based on the results, we conclude that *M. thermaggregans* is a suitable organism for CH_4_ production. Results on the comparative performance of *M. thermaggregans* and *M. marburgensis* indicated that both organisms are equally suited to be employed as CH_4_ cell factories. From a bioprocess technological point of view, *M. thermaggregans* required an adaption period to be able to grow at a high agitation speeds, whereas *M. marburgensis* did not.

## Electronic supplementary material

ESM 1 (PDF 229 kb)
