# Supplementary material for: Physiology and methane productivity of Methanobacterium thermaggregans
Source: Appl Microbiol Biotechnol. 2018 Jun 29;102(17):7643–56. doi: 10.1007/s00253-018-9183-2 (PMC6097776; doi:10.1007/s00253-018-9183-2)
Supplement: Supplementary file 1 — (PDF 229 kb) [file 253_2018_9183_MOESM1_ESM.pdf]

Applied Microbiology and Biotechnology

Physiology and methane productivity *of Methanobacterium thermaggregans*

Lisa-Maria Mauerhofer[1], Barbara Reischl[1,2], Tilman Schmider[1], Benjamin Schupp[1], Kinga Nagy[1,3], Patricia Pappenreiter[4], Sara Zwirtmayr[4], Bernhard Schuster[3], Sébastien Bernacchi[2], Arne H. Seifert[2], Christian Paulik[4], Simon K.-M. R. Rittmann[1,*]

[1]Archaea Physiology & Biotechnology Group, Archaea Biology and Ecogenomics Division, Department of Ecogenomics and Systems Biology, Universität Wien, Wien, Austria

[2]Krajete GmbH, Linz, Austria

[3]Institute of Synthetic Bioarchitectures, Department of Nanobiotechnology, University of Natural Resources and Life Sciences, Wien, Austria

[4]Institute for Chemical Technology of Organic Materials, Johannes Kepler Universität Linz, Linz, Austria

[*]Corresponding author:

Dr. Simon K.-M. R. Rittmann

Archaea Physiology & Biotechnology Group

Archaea Biology and Ecogenomics Division

Department of Ecogenomics and Systems Biology

Universität Wien

Althanstraße 14

1090 Wien

Austria

Tel.: +43-4277-76513

eFax.: +43-4277-876513

email: simon.rittmann@univie.ac.at

Table S1: ANOVA for Response Surface Reduced Quadratic Model x_{max}

x_{max}= Log10((-35.24755687+(0.762155327* Temperature)+ (3.190981698* pH)+ (-0.00623276*Temperature^2)+ (-0.211348003* pH^2))						
Analysis of variance table [Partial sum of squares - Type III]						
	Sum of		*Mean*	*F*	*p-value*	
Source	*Squares*	*df*	*Square*	*Value*	*Prob > F*	
Model	*0.894493019*	*4*	*0.223623255*	*22.65421373*	*< 0.0001*	*significant*
A-Temperature	*0.087490079*	*1*	*0.087490079*	*8.863205926*	*0.0089*	
B-pH	*0.165839363*	*1*	*0.165839363*	*16.8004011*	*0.0008*	
A^2	*0.625867049*	*1*	*0.625867049*	*63.40362906*	*< 0.0001*	
B^2	*0.037701654*	*1*	*0.037701654*	*3.819376161*	*0.0684*	
Residual	*0.15793848*	*16*	*0.009871155*			
Lack of Fit	*0.064093756*	*5*	*0.012818751*	*1.502548646*	*0.2660*	*not significant*
Pure Error	*0.093844724*	*11*	*0.008531339*			
Cor Total	*1.052431498*	*20*				
Std. Dev.	*0.099353686*		*R-Squared*	*0.84992992*		
Mean	*-0.149218223*		*Adj R-Squared*	*0.812412399*		
C.V. %	*66.58281013*		*Pred R-Squared*	*0.705255087*		
PRESS	*0.310198831*		*Adeq Precision*	*16.83061995*		
	Coefficient		*Standard*	*95% CI*	*95% CI*	
Factor	*Estimate*	*df*	*Error*	*Low*	*High*	*VIF*
Intercept	*0.024646613*	*1*	*0.033546624*	*-0.046469053*	*0.095762279*	
A-Temperature	*0.071120654*	*1*	*0.023889129*	*0.020477962*	*0.121763346*	*1.029638993*
B-pH	*0.092843863*	*1*	*0.022651313*	*0.044825225*	*0.140862501*	*1.069259789*
A^2	*-0.155818999*	*1*	*0.019568762*	*-0.197302922*	*-0.114335077*	*1.03079817*
B^2	*-0.03381568*	*1*	*0.01730302*	*-0.070496445*	*0.002865084*	*1.091888669*

Table S2: ANOVA for Response Surface Reduced Quadratic *Model* $r_{(x)\ max}$

r(x) max= Log10(-43.55859742+ (0.894196753 Temperature)+ (4.532229376 * pH)+ (-0.007334806*Temperature^2)+ (-0.304943494* pH^2)*

Analysis of variance table [Partial sum of squares - Type III]

Source	Sum of Squares	df	Mean Square	F Value	p-value Prob > F	
Model	1.21513411	4	0.303783527	15.31034354	< 0.0001	significant
A-Temperature	0.084997108	1	0.084997108	4.283757371	0.0550	
B-pH	0.21295129	1	0.21295129	10.73250231	0.0048	
A^2	0.86675941	1	0.86675941	43.68368639	< 0.0001	
B^2	0.078487926	1	0.078487926	3.955701988	0.0641	
Residual	0.317467497	16	0.019841719			
Lack of Fit	0.069843725	5	0.013968745	0.620522797	0.6876	not significant
Pure Error	0.247623772	11	0.022511252			
Cor Total	1.532601606	20				
Std. Dev.	0.140860635		R-Squared	0.792857129		
Mean	0.256961055		Adj R-Squared	0.741071411		
C.V. %	54.81789264		Pred R-Squared	0.660030587		
PRESS	0.521037668		Adeq Precision	12.93314562		
Factor	Coefficient Estimate	df	Standard Error	95% CI Low	95% CI High	VIF
Intercept	0.471279976	1	0.047561383	0.370454348	0.572105604	
A-Temperature	0.070100064	1	0.033869281	-0.001699605	0.141899732	1.029638993
B-pH	0.105208185	1	0.032114342	0.037128821	0.17328755	1.069259789
A^2	-0.183370154	1	0.027743996	-0.242184798	-0.12455551	1.03079817
B^2	-0.048790959	1	0.024531696	-0.100795831	0.003213913	1.091888669

Table S3: ANOVA for Response Surface Reduced Quadratic Model MER_{max}

MER_{max}= Log10(-37.71197957+ (0.651467497 Temperature)+ (5.199576927*pH)+ (-0.005140405*Temperature^2)+ (-0.354136403*pH^2)*

Analysis of variance table [Partial sum of squares - Type III]

	Sum of		Mean	F	p-value	
Source	Squares	df	Square	Value	Prob > F	
Model	1.12140412	4	0.28035103	43.40916293	< 0.0001	significant
A-Temperature	0.518242887	1	0.518242887	80.24400674	< 0.0001	
B-pH	0.179778162	1	0.179778162	27.83660023	< 0.0001	
A^2	0.425712088	1	0.425712088	65.91666659	< 0.0001	
B^2	0.105853507	1	0.105853507	16.39020952	0.0009	
Residual	0.103333402	16	0.006458338			
Lack of Fit	0.028823289	5	0.005764658	0.851042014	0.5420	not significant
Pure Error	0.074510113	11	0.006773647			
Cor Total	1.224737522	20				
Std. Dev.	0.080363783		R-Squared	0.915628124		
Mean	1.75082873		Adj R-Squared	0.894535155		
C.V. %	4.590042511		Pred R-Squared	0.850831856		
PRESS	0.182691823		Adeq Precision	24.09421267		
	Coefficient		Standard	95% CI	95% CI	
Factor	Estimate	df	Error	Low	High	VIF
Intercept	1.914966472	1	0.027134711	1.857443453	1.97248949	
A-Temperature	0.173094398	1	0.019323096	0.132131264	0.214057531	1.029638993
B-pH	0.096666915	1	0.018321869	0.057826289	0.135507542	1.069259789
A^2	-0.128510129	1	0.015828499	-0.162065048	-0.094955209	1.03079817
B^2	-0.056661824	1	0.013995818	-0.086331634	-0.026992015	1.091888669

Table S4: ANOVA for Response Surface Reduced Quadratic Model $MER_{max\ (drum\text{-}type\ gas\ meter)}$

$$MER_{max\ (drum\text{-}type\ gas\ meter)} = Log10(-34.59247361 + (0.750491255*Temperature) + (3.550113117*pH) + (-0.00597897*Temperature^2) + (-0.241172243*pH^2)$$

Analysis of variance table [Partial sum of squares - Type III]

Source	Sum of Squares	df	Mean Square	F Value	p-value Prob > F	
Model	1.100703308	4	0.275175827	26.22774156	< 0.0001	significant
A-Temperature	0.471330906	1	0.471330906	44.923805	< 0.0001	
B-pH	0.092877263	1	0.092877263	8.852379516	0.0089	
A^2	0.575935754	1	0.575935754	54.89397194	< 0.0001	
B^2	0.049092907	1	0.049092907	4.679175856	0.0460	
Residual	0.167868561	16	0.010491785			
Lack of Fit	0.088179593	5	0.017635919	2.43440354	0.1016	not significant
Pure Error	0.079688968	11	0.007244452			
Cor Total	1.268571869	20				

Std. Dev.	0.102429415	R-Squared	0.867671225
Mean	1.785148567	Adj R-Squared	0.834589032
C.V. %	5.737865011	Pred R-Squared	0.728077663
PRESS	0.344953027	Adeq Precision	19.49840082

Factor	Coefficient Estimate	df	Standard Error	95% CI Low	95% CI High	VIF
Intercept	1.946061369	1	0.034585139	1.872744149	2.019378589	
A-Temperature	0.165074236	1	0.024628674	0.11286378	0.217284692	1.029638993
B-pH	0.069480686	1	0.023352538	0.019975517	0.118985854	1.069259789
A^2	-0.149474252	1	0.020174559	-0.192242407	-0.106706096	1.03079817
B^2	-0.038587559	1	0.017838676	-0.076403863	-0.000771255	1.091888669

Table S5: ANOVA for Response Surface Reduced Quadratic Model cum. CH_4 production

cum. CH4 production= (-139254.2929+(2307.001118 Temperature)+ (19715.7902* pH)+ (-18.85017552* Temperature^2)+ (-1370.515378* pH^2)*

Analysis of variance table [Partial sum of squares - Type III]

Source	Sum of Squares	df	Mean Square	F Value	p-value Prob > F	
Model	8446243.846	4	2111560.961	8.265314498	0.0008	significant
A-Temperature	874877.6436	1	874877.6436	3.424546581	0.0828	
B-pH	860031.46	1	860031.46	3.366433943	0.0852	
A^2	5724691.389	1	5724691.389	22.4082447	0.0002	
B^2	1585374.735	1	1585374.735	6.205655919	0.0241	
Residual	4087560.781	16	255472.5488			
Lack of Fit	946365.287	5	189273.0574	0.662806131	0.6593	not significant
Pure Error	3141195.494	11	285563.2267			
Cor Total	12533804.63	20				
Std. Dev.	505.4429234		R-Squared	0.673877095		
Mean	1528.420456		Adj R-Squared	0.592346368		
C.V. %	33.06962566		Pred R-Squared	0.397662669		
PRESS	7549578.421		Adeq Precision	9.466851798		
Factor	Coefficient Estimate	df	Standard Error	95% CI Low	95% CI High	VIF
Intercept	2160.420457	1	170.6620487	1798.633076	2522.207839	
A-Temperature	224.9002801	1	121.5313878	-32.7347529	482.5353131	1.029638993
B-pH	211.4299759	1	115.234232	-32.85568309	455.7156349	1.069259789
A^2	-471.254388	1	99.55234348	-682.2959284	-260.2128476	1.03079817
B^2	-219.2824605	1	88.02581397	-405.8888499	-32.67607103	1.091888669
